# The comparison of krill oil extracted through ethanol–hexane method and subcritical method

**DOI:** 10.1002/fsn3.914

**Published:** 2019-01-28

**Authors:** Weiwei Sun, Bowen Shi, Changhu Xue, Xiaoming Jiang

**Affiliations:** ^1^ College of Food Science and Engineering Ocean University of China Qingdao China; ^2^ Laboratory for Marine Drugs and Bioproducts of Qingdao National Laboratory for Marine Science and Technology Qingdao China

**Keywords:** astaxanthin, ethanol–hexane extraction, krill oil, phospholipid, subcritical extraction

## Abstract

This study aimed to develop a safe method EH (ethanol–hexane) to extract two kinds of krill oil (KO) simultaneously and analyze their composition. Meanwhile, subcritical butane and subcritical butane‐dimethyl ether extraction were used to extract KO for analysis comparison. Folch method was used to extract total lipids. When the volume ratio of ethanol to hexane is 4:6, the separation effect of ethanol layer and hexane layer is best. At this condition, the EH method yielded similar amount of lipids (up to 97. 72% of total lipids) with subcritical butane extraction method (97.60%). The recovery rate of ethanol and hexane was 83.6% and 86.86%, respectively. KO in hexane layer and extracted by the subcritical butane method are abundant in astaxanthin (910 and 940 mg/kg respectively), while KO in the ethanol layer had the highest phospholipid (PL) content (47.34%), *n*−3 polyunsaturated fatty acids (PUFA) content (45.51%), and the lowest fluorine content (11.17 μg/g), making it a potential candidate in the nutraceutical and antioxidant industry.

## 1 INTRODUCTION

Antarctic krill (*Euphausia superba*) is the predominant prey in the Southern Ocean owing to its rich biomass (Auerswald, Meyer, Teschke, Hagen, & Kawaguchi, 2015; Wu et al., 2015), and 70% of which are distributed between longitudes 0° and 90°W (Atkinson et al., 2008). It comprises 77.9%–83.1% moisture, 11.9%–15.4% protein, 0.4%–3.6% lipids, and approximately 2% chitin (Grantham, 1977). It is also a rich and potential alternative source of long‐chain omega‐3 polyunsaturated fatty acids (LC‐ω‐3 PUFAs; Xi et al., 2016). Krill oil (KO), with abundant eicosapentaenoic acid (C20:5 EPA), docosahexaenoic acid (C22:6 DHA), as well as antioxidant (Lu et al., 2017), has attracted increasing attention. EPA and DHA in KO are for the synthesis of both phospholipids (PLs) and triglycerides (TGs), while in fish oil (FO) are primarily just in the form of TGs (Liu et al., 2014; Rossmeisl et al., 2012). Some studies show that plasma levels of EPA and DHA increased with the consumption of KO rather than FO (Costanzo et al., 2016; Ramprasath, Eyal, Zchut, & Jones, 2013; Ramprasath, Inbal, Sigalit, & Jones Peter, 2014). In addition, the unique and particular amphiphilic nature of PLs brings emulsification properties to KO, thus improving the bioavailability of EPA and DHA (Schuchardt et al., 2011). Moreover, compared with FO, KO has a significant amount of astaxanthin, a potent and naturally occurring antioxidant, which can not only reduce the oxidation of KO (Hussein et al., 2007), but also provides health‐promoting properties such as reducing the incidence of inflammation, cancer, diabetes, immune function, and hyperlipidemia (Feng et al., 2018; Kim et al., 2016; Sun et al., 2017).

Organic solvents and supercritical carbon dioxide (SC‐CO2) are most commonly used for industrial oil extraction (including KO; Bruheim et al., 2015; Gigliotti, Davenport, Beamer, Tou, & Jaczynski, 2011; Sahena et al., 2009; Xie et al., 2017). Though the properties of components in KO separated via the SC–CO_2_ method can be improved in certain cases, high capital costs for batch extraction and engineering hardware technology should be considered (Friedrich & Pryde, 1984; Gigliotti et al., 2011). These years subcritical solvents are becoming popular to extract oil since this method is easier than the supercritical method regarding its application in industries with a higher productivity (Xu, Han, Zhou, Wu, & Ding, 2016). What's more, subcritical butane extraction has safe, efficient, and environmental compatibility. The extraction is a continuous counter current process in which the solvent can be removed completely by system depressurization (Guan, Jin, Li, Huang, & Liu, 2018). Organic solvent extraction could extract KO more simply without expensive instruments (Xie et al., 2017). Traditionally, KO is extracted via a two‐step solvent extraction, using acetone and ethanol (Beaudoin & Martin, 2004). However, these two separate extraction steps are laborious and inefficient. The process to extract KO has been improved via one‐step extraction with isochoric ethanol and acetone (Gigliotti et al., 2011), using freeze‐dried krill as raw material, making oil extraction more efficient. Though freeze‐drying can preserve food quality of products, it is usually used in small‐scale and biopharmaceutical industries because of high expenses for equipment and energy consumption (Tang, Tian, Lee, & Row, 2012). Air convective dryers with hot air are commonly used, while can result in severe damage to food quality, such as nutrient loss, bad taste, and color deterioration (Maskan, 2001). Moreover, serious environmental issues have arisen owing to the use of environmentally unfriendly solvents.

In this study, mixture of environmentally friendly solvents ethanol and hexane was applied as solvents to obtain two kinds of KO. The effects of different conditions (ethanol/hexane ratio, time, temperature, shrimp/solvent ratio) on the lipid yield were investigated. It is reported that the extrusion of krill meal prior to oil extraction could promote lipid yield when using *n*‐hexane as the extraction solvent (Yin et al., 2015). Hence, before the experiment, Antarctic krill were extruded until its water content reduced to 60%. This method was compared with the subcritical extraction method. The quality of KO extracted via EH and subcritical method was measured: PL, fatty acids (FA), and minor components including astaxanthin, fluorine, ash, and arsenic in the extracted KO were analyzed.

## 2 MATERIALS AND METHODS

### 2.1 Materials

Antarctic krill were obtained from China National Fisheries Co., Ltd. Then, the shrimp meal was obtained after the process of Microwave thawing, heating at 95°C for 5 min, and centrifugation, followed by freezing. Frozen Antarctic krill were then delivered to our laboratory and stored at −30°C until use. Prior to the experiments, the krill were crushed using a hammer crusher and then stored in a low‐temperature (−30°C) warehouse.

### 2.2 Lipid extraction

When ethanol and hexane were used as extraction solvents, the following experiments were conducted:

10 g of frozen Antarctic krill was weighed, and KO was extracted using EH (ethanol/hexane = 4:6). After extraction, the filtrate was stratified, and the upper hexane layer and the lower ethanol layer were separated. KO in the upper and lower layers was obtained via rotary evaporation and then collected in a glass vessel for analysis. The optimization experiments were shown in Supporting Information Data S1.

$$\text{Lipid extraction efficiency}\%=\frac{\text{The KO extracted by EH}\;}{\text{The KO extracted by Folch}\;}\times 100\%$$

A pilot‐scale subcritical extraction unit purchased from Henan Subcritical Bio Technology Co., Ltd. (Anyang, Henan, China) was used to conduct the subcritical extraction. The experiment was performed at 30℃ for 1 hr at a pressure range of 0.3–0.8 MPa and was repeated four times with butane and butane‐dimethyl ether as solvent (Xu et al., 2016).

For comparison, Antarctic krill oil was assessed via Folch method after the Antarctic krill cells were thoroughly homogenized (Folch, Lees, & Sloane Stanley, 1957).

### 2.3 Solvent recovery rate

After lipid extraction through EH method, the volume of recycled ethanol and hexane was recorded, respectively, to calculate the recovery rate. That rate was determined gravimetrically based on the following formula:

$$\text{Recovery rate}=\frac{a}{b}\times 100\%$$

where *a* is the volume of the recycled ethanol or hexane(v) and *b* is the previously added ethanol or hexane(v).

### 2.4 Determination of PLs in extracted KO via nuclear magnetic resonance (NMR) analysis

The PL content was determined using ^31^P NMR analysis, as previously described by Li et al. (Li, 2014) with slight modifications. All NMR experiments were conducted with a Bruker Avance Spectrometer 500 (Bruker, Germany) operating at 243 MHz and 25°C. Lipid samples (5 mg) dissolved in 900 μl of CDCl_3_ and 100 μl of triphenylphosphate (TPP, 100 mM) as an internal standard were placed in NMR tubes. Typical chemical shift values obtained are summarized as follows: δ 17.1 (TPP), 0.56 (phosphatidylcholine [PC]), and 0.6 (phosphatidylethanolamine [PE]). The yields of PC and PE were calculated in accordance with the peak area ratios relative to that of the TPP reference.

### 2.5 Determination of astaxanthin in extracted KO via high‐performance liquid chromatography (HPLC)

An HPLC system (LC‐20AT, Shimadzu, Kyoto, Japan) equipped with an ultraviolet detector (SPD‐20A, Shimadzu, Kyoto, Japan) and YMC‐Carotenoid‐C30 (4.6 × 250 mm, 5 μm), Japan YMC Co., Ltd. was used to analyze astaxanthin based on the method described by Rao, Baskaran, Sarada, and Ravishankar (2013) with slight modifications. Briefly, the extracted KO was dissolved in 3 ml of dichloromethane. After filtration with a 0.22‐micron filter, 0.5 ml of the filtrate was placed into liquid vials. The astaxanthin was analyzed via HPLC. Astaxanthin was detected at 476 nm, and the measured quantity was based on the peak area of total astaxanthin.

### 2.6 Composition analysis of FA via gas chromatography (GC)

Thin‐layer chromatography (TLC) plates were used to isolate TG and PL in the krill oil samples, and the developing solvent was hexane:diethyl ether:acetic acid (80:20:1, v:v:v). The bands of TG and PL were scraped off, extracted with Folch method, and dried with nitrogen.

The lipids were converted to fatty acid methyl esters (FAMEs) through alkali/catalyzed transesterification/esterification, in accordance with Cinzia Chiappe's method Chiappe et al. (2016) with slight modifications. Briefly, 50 μl of KO was dissolved in 2.5 ml of methanol‐KOH, in a water bath at 65°C for 3 min. Three milliliters of 10% methanol–sulfuric acid was added, and the mixture was incubated in a water bath at 65°C for 30 min. After cooling to room temperature (25°C), 1 ml of *n*‐hexane was added to dilute the upper organic layer after centrifugation for 5 min at 11,180 g at 4°C and used for analysis; the FAMEs that were recovered were analyzed with an Agilent Technologies 7820A GC equipped with a HP‐INNOWax quartz capillary column (30 × 0.32 mm; coating thickness 0.25 μm). The FA contents were expressed as a weight percentage (%, w/w) of the total content of FAs detected with 14–22 carbon‐atom chain lengths (Yin et al., 2015).

### 2.7 Determination of fluoride content in extracted KO with a fluoride ion‐sensitive electrode (ISE)

Fluoride content in KO was determined using a fluoride ISE in accordance with a standard addition method reported by Trombella, Caputi, Musso, Ribeiro, and Ryan (2003) First, the sample was pretreated in accordance with the method of Malde, Bjorvatn, and Julshamn (2001)) with a slight modification. Hundred milligrams of KO was accurately weighed and put into a beaker and then covered with 10 ml 0.1 M perchloric acid to dissolute for 2 hr. After dissolution, the sample solution was transferred into a 50‐ml volumetric flask. Total ionic strength adjustment buffer (TISAB) solution (25 ml) was added to the flask and then diluted with deionized water to the required volume. The contents were shaken well, allowed to stand for 30 min, and poured into a 100‐ml plastic beaker. Thereafter, the method described by Yin et al. (2017) was followed to determine fluoride content.

### 2.8 Determination of arsenic content in extracted KO via inductively coupled plasma mass spectrometry (ICP‐MS)

For microwave digestion treatment, 0.3 g of KO was digested with 10 ml of HNO_3_. The sample was allowed to stand for approximately 10 min to eliminate the gases generated initially by the mixture and to avoid an excessive increase in pressure in the digestion process.

For ICP‐MS analysis, after digestion, the solutions (approximately 11 ml) were diluted to a final volume of 100 ml, filtered with a 0.45 μl membrane into clean polyethylene flasks, and stored in a refrigerator at 4°C until use.

Thereafter, the method of López, Garcia, Morito, and Vidal (2003) was followed to determine the arsenic content.

### 2.9 Determination of free fatty acids in extracted KO via the colorimetric method

Free fatty acid (FFA) content of each oil was determined in accordance with Véroniquej, Virginia, and Jamesk (2008) with slight modification. Reagents and samples were equilibrated to 20 ± 3°C; 20 mg of KO was dissolved in 3 ml of *n*‐heptane and stirred for 1 min. One milliliter of copper reagent was then added, the mixture was stirred for 2 min, and the developed color was measured using a colorimeter with a 715‐nm filter after incubation at room temperature (25°C) for 10 min. The results were compared to a standard curve plotted from samples of oleic acid.

### 2.10 Statistical analysis

The oil extraction experiments were performed in triplicate (*n* = 3). For each triplicate, at least three measurements were performed, and the results were shown as the mean ± standard deviation. Statistical analysis was conducted using the statistical software SPSS version 20.0. The results were statistically evaluated by one‐way analysis of variance (ANOVA) using Tukey's test. Significant differences between means were assessed at *p* < 0.05.

And a supervised OPLS‐DA method was then applied to sharp the classification effect of KO extracted through different methods (Wang et al., 2016).

## 3 RESULTS AND DISCUSSION

### 3.1 Effect of different conditions on lipid yield via the EH method

Supporting Information Figure S1 showed that ethanol and hexane destroyed the outer structure of shrimp powder, thus increasing the dissolution of oil.

**Figure 1:**
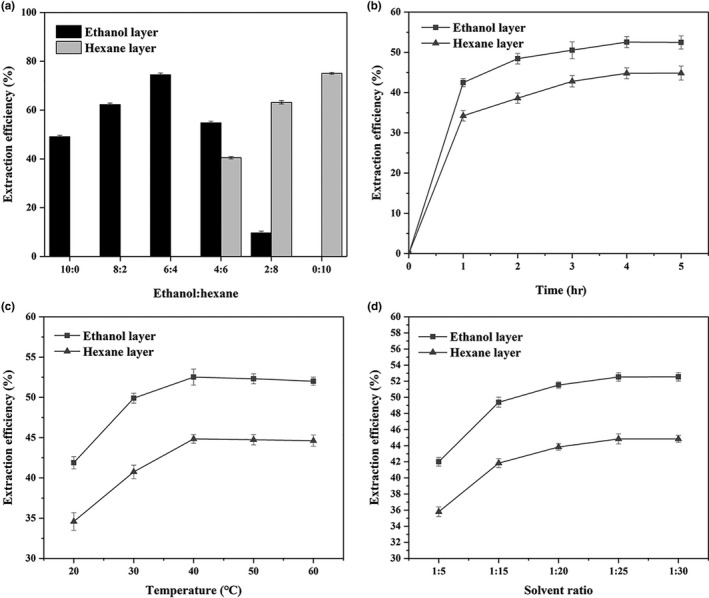
(a) Effect of different ethanol–hexane ratios on lipid extraction efficiency of ethanol and hexane layers; (b) effect of different extraction times on lipid extraction efficiency of ethanol and hexane layers; (c) effect of different temperatures on lipid extraction efficiency of ethanol and hexane layers; (d) effect of different solvent ratios on lipid extraction efficiency of ethanol and hexane layers. The lipid extraction efficiency is the sum of both in ethanol and hexane layers

As shown in Figure 1a, when the volume of ethanol was higher than hexane, the filtrate did not stratify. As the proportion of ethanol in the extraction solvent decreased, lipid extraction efficiency increased. When the volumetric ratio of ethanol/hexane was 6:4, lipid extraction efficiency increased up to 74.49%. When the volume of hexane was higher than ethanol, after standing for 30 min, the filtrate stratified into two layers. The total lipid extraction efficiency can approach 95.23% (54.77% for ethanol layer and 40.46% for hexane layer) at a volumetric ratio of ethanol/hexane 4:6.

The effect of reaction time on lipid extraction efficiency of ethanol and hexane layers was evaluated and presented in Figure 1b. The lipid extraction efficiency of both layers increased gradually with time. The highest lipid extraction efficiency of 52.53% (ethanol layer) and 44.80% (hexane layer) was achieved at 4 hr, with no significant increase thereafter.

Figure 1c showed that the lipid extraction efficiency of both layers was further enhanced with an increase in temperature from 20 to 40°C, which resulted in a prominent increase in lipid extraction efficiency from 76.47% to 97.37% (52.52% of ethanol layer and 44.85% of hexane layer). However, a slight reduction was observed beyond 40°C.

As shown in Figure 1d, lipid extraction efficiency of both layers gradually increased with addition of solvent until equilibrium was attained. A solvent ratio of 1:25 served as the optimum value to obtain a lipid extraction efficiency of 97.38% (52.53% of ethanol layer and 44.85% of hexane layer), beyond which there was no significant change in efficiency.

Meanwhile, the recovery rate of ethanol was 83.6%, and that of hexane was 86.86%.

### 3.2 Comparison of different kinds of KO composition

Lipid yield (%), water (%), astaxanthin (%), FFA (%), and ash (%) in KO extracted through different methods are shown in Table 1. Regarding lipid yield, Folch method yielded up to 8.34% (KO/krill with 60% moisture, ω%) in total. 8.15% of KO (3.77% in the hexane layer and 4.38% in the ethanol layer) was obtained using the EH method, accounting for 97.72% of total lipids, subcritical butane extracted for 97.60% of total lipids, while the subcritical butane‐dimethyl ether method yielded a much lower amount of lipids (3.12%) than the other extraction methods. Since the subcritical dimethyl ether can simultaneously extract water and oil (Fang et al., 2018), the separation process of oil and water would affect the yield of oil owing to hydrophilic–lipophilic PLs in Antarctic KO.

**Table 1:** Comparison of lipid yield (%) in frozen krill and water (%), astaxanthin (%), FFA (%), and ash (%) in KO extracted using different methods

	Hexane layer	Ethanol layer	Butane‐dimethyl	Butane	Folch
Lipid yield (%)	3.77 ± 0.32^b^	4.38 ± 0.45^c^	3.12 ± 0.78^a^	8.14 ± 1.1^d^	8.34 ± 0.3^e^
Water (%)	1.78 ± 0.23^b^	10.35 ± 0.45^c^	14.46 ± 0.33^e^	12.33 ± 0.43^d^	1.76 ± 0.32^a^
Astaxanthin (mg/kg)	910 ± 0.11^d^	80 ± 0.12^a^	440 ± 0.41^b^	940 ± 0.22^e^	690 ± 0.23^c^
Free fatty acids (%)	9.26 ± 0.23^a^	32.68 ± 0.24^e^	24.18 ± 0.15^d^	15.54 ± 0.23^b^	16.57 ± 0.5^c^
Ash (%)	19.64 ± 0.2^a^	51.25 ± 0.31^e^	32.2 ± 0.11^b^	35 ± 0.32^c^	39 ± 0.24^d^

Note

Values are means ± standard deviation and are expressed as mass%. Different superscript letters in a row indicate significant differences for individual component (*p* < 0.05).

Water content in all KO samples varied from 1.76% to 14.46%. Regarding the EH method, high water content (10.35%) was detected in KO from the ethanol layer, and markedly low water content (1.78%) was detected in KO from the hexane layer. Folch method yielded the lowest water content (1.76%). The KO extracted through subcritical methods contained higher water, 14.46% water content via the subcritical butane‐dimethyl ether method and 12.33% water content via the subcritical butane method. It is reported that when at proper water activity, water may act as an antioxidant by hydrating or diluting catalytic metal oxides; however, with an increase in water content, it may serve as a prooxidant via solubilization of these catalysts (Ghnimi, Budilarto, & Kamal‐Eldin, 2017). In this way, through the water content, we can conclude that the KO extracted by the subcritical method is more susceptible to oxidation than the KO extracted by the EH method.

The highest astaxanthin yield (up to 940.00 mg/kg) was achieved via the subcritical butane method. In the EH method, KO from the hexane layer yielded a similar proportion (910.00 mg/kg), while KO from the ethanol layer yielded the lowest amount of astaxanthin (80.00 mg/kg). According to Sánchez‐Camargo, Martinez‐Correa, Paviani, and Cabral (2011) the most common method for extracting carotenoids from crustaceans is through nonpolar solvents (Sánchez‐Camargo, Martinez‐Correa, Paviani, & Cabral, 2011). The results in Table 1 also showed that astaxanthin was more soluble in nonpolar solvents rather than polar solvents.

**Figure 2:**
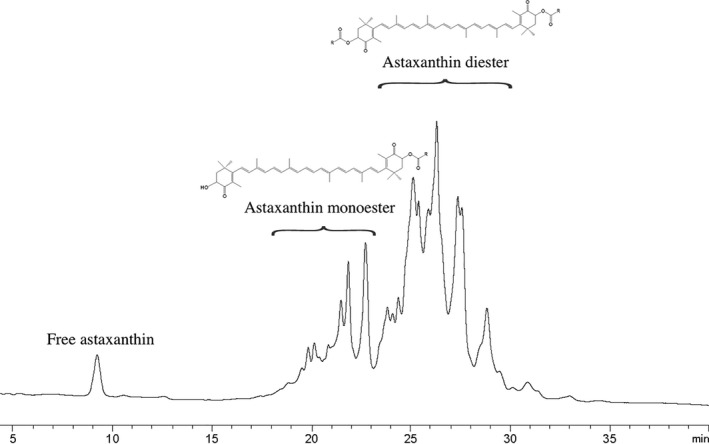
HPLC‐DAD chromatography of astaxanthin and astaxanthin esters in KO

Figure 2 showed the distribution of free astaxanthin and astaxanthin esters in KO. The free astaxanthin accounted for a small part. In particular, the astaxanthin diester was significantly more than the astaxanthin monoester, which was exactly the opposite distribution of astaxanthin extracted from *Haematococcus pluvialis* (Maoka, Katsuyama, Kaneko, & Matsuno, 1985; Miao, Lu, Li, & Zeng, 2006) achieved similar results.

Due to the long storage time of Antarctic krill, the content of FFA shown in Figure 1 was generally high. KO extracted by the subcritical butane method contained similar FFA content to KO extracted by Folch method (15.54% and 16.57%, respectively). However, in the EH method, the hexane and ethanol layers did not distribute evenly, and FFA content of KO in the ethanol layer was 32.68%. A similar trend was observed with ash content in KO. In the ethanol layer, ash content of KO approached 51.25%; hexane layer just for 19.64%.

**Figure 3:**
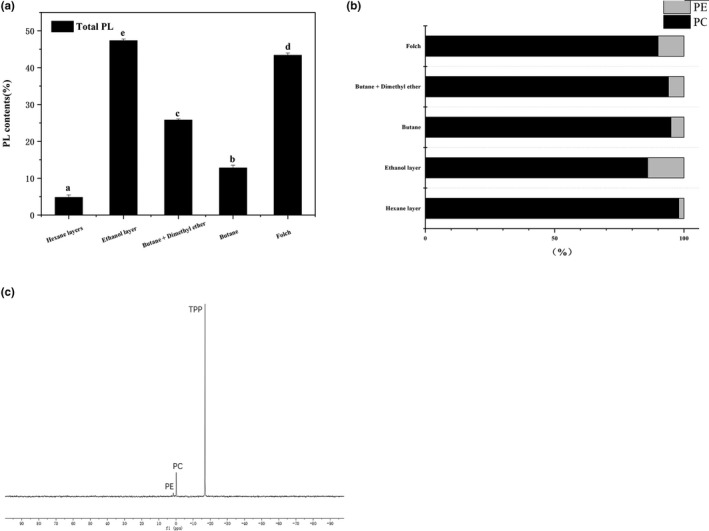
(a) Comparison of PL content in KO extracted through different methods. (b) Comparison of PC/PE ratio. (c) NMR analysis of PC and PE in KO. Different letters on the top of data bars indicate significant differences (Tukey's test, *p* < 0.05) between mean values (±*SD*,* n* = 3)

Phospholipid (PL) content presented as wt% of extracted KO (Figure 3). As shown in Figure 3a, as for the EH method, PL content of KO in the ethanol layer (47.34%) was higher than that using Folch method (43.37%). On the contrary, the PL content of KO in the hexane layer was the lowest, accounting for 4.81% of KO. Furthermore, the subcritical butane‐dimethyl ether extraction method yielded a higher phospholipid content (25.78%) than the subcritical butane extraction method (12.82%) because subcritical butane allowed for selective extraction of phospholipids, thereby potentially decreasing the yield of extracted phospholipids (Xu et al., 2016).

Phosphatidylcholine (PC) and phosphatidylethanolamine (PE) were the only two types of PLs detected in all KO samples, and the relative proportions of PC and PE are shown in Figure 3b. In general, PC was the most abundant PL, ranging from 86% to 98%, whereas PE constituted a considerably lower proportion (2%–14%). The results showed that PC is the most abundant PL in KO, thereby rendering KO an optimal source of natural PC.

Table 2 listed the FA composition and content in extracted KO, with the FAs grouped by type, as C14:0, C16:0, C16:1, C18:1, C20:1, C20:5 (EPA), and C22:6 (DHA) were the primary FAs in the oil, concurrent with previous findings. (Fricke, Gercken, Schreiber, & Oehlenschläger, 1984; Phleger, Nelson, Mooney, & Nichols, 2002) Essentially, *n*−3 PUFA (mainly EPA and DHA in KO) were particularly abundant, accounting for more than 23% of the total FA in all samples.

**Table 2:** Comparison of fatty acid composition and content in KO extracted through different methods

Fatty acid (%)	Hexane layer	Ethanol layer	Butane‐dimethyl	Butane	Folch
C14:0	14.7	5.03	7.19	8.33	9.31
C16:0	28.56	27.81	26.51	18.25	27.61
C16:1	6.52	4.06	4.62	5.59	5.52
C17:0	2.45	–	–	1.58	1.62
C17:1	–	–	1.64	–	–
C18:0	13.32	7.81	8.26	12.58	10.68
C18:1 (*n*−9)	9	6.44	7.15	7.04	7.57
C18:2 (*n*−6)	1.67	1.99	2.29	3.45	1.89
C18:3 (*n*−6)	–	1.35	1.22	2.14	–
C18:3 (*n*−3)	3.13	2.87	2.93	2.9	2.79
C20:1 (*n*−9)	–	–	–	1.11	–
C20:5 (*n*−3)	12.74	28.47	24.43	17.67	21.4
C22:6 (*n*−3)	7.92	14.17	13.77	9.36	11.62
SFA	59.03	40.65	41.96	40.74	49.22
MUFA	15.52	10.5	13.41	13.74	13.09
PUFA	25.46	48.85	44.64	35.52	37.7
PUFA (*n*−3)	23.79	45.51	41.13	29.93	35.81

Note

MUFA: monounsaturated fatty acids; PUFA: polyunsaturated fatty acids; SFA: saturated fatty acid.

Additionally, FA composition, especially *n*−3 PUFA, exhibited considerable differences among KO samples extracted via different methods. Greater amounts of omega‐3 (*n*−3) FAs were observed in the KO from the ethanol layer (up to 45.51% of FAs), while KO from the hexane layer had the lowest level of *n*−3 PUFAs (23.79%). Moreover, *n*−3 PUFA content in KO extracted through the subcritical butane‐dimethyl ether method (up to 41.13%) was higher than that obtained via the subcritical butane method. The results showed the same trend as that of PLs (Figure 3a), since the *n*−3 PUFA occurs naturally in KO mostly in the form of PL (Haider, Majeed, Williams, Safdar, & Zhong, 2017).

**Figure 4:**
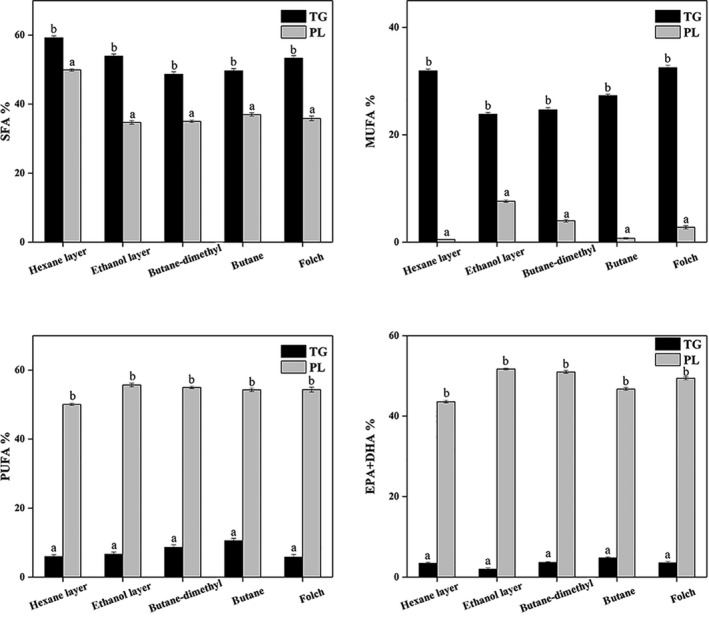
Comparison of major fatty acids associated with PL and TG in krill oil extracted through different methods. *Different letters on the top of data bars indicate significant differences (Tukey's test, *p* < 0.05) between mean values (±*SD*,* n* = 3) within a lipid class

Figure 4 showed the comparison of the major FA (SFA, MUFA, PUFA, EPA + DHA) contents of PL and TG in KO extracted through different methods. There was a significant difference (*p* < 0.05) between PL and TG in the composition of the FA. The percentage of PUFA and EPA + DHA in PL was much higher (*p* < 0.05) than in TG, and the percentage of SFA and MUFA was lower than TG. In addition, EPA and DHA had a higher association with PL. Specifically, 43.58%–51.7% of EPA + DHA was observed in PL, while a significantly lower level was detected in TG (1.98%–4.79% of EPA + DHA), indicating that EPA and DHA were predominant in PL and contributed to a much higher level of total PUFA in PL than TG. Therefore, it can be concluded that higher PL extraction efficiency can increase the PUFA content in krill oil. EPA and DHA are biologically active *n*−3 polyunsaturated fatty acids that are associated with health benefits, such as reducing the risk of cardiovascular disease (cardiovascular disease). Therefore, esterification of EPA and DHA into PL in KO may be important for human health.

**Figure 5:**
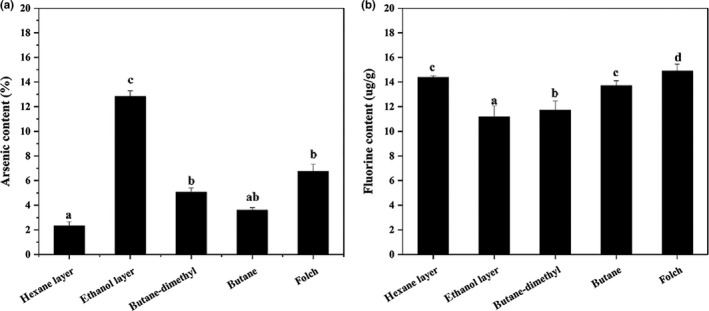
Arsenic (a) and fluorine (b) content in KO extracted through different methods. Different letters on the top of data bars indicate significant differences (Tukey's test, *p* < 0.05) between mean values (±*SD*,* n* = 3)

Arsenobetaine is reportedly the major arsenical in the Antarctic krill, with a concentration of 1.9 μg/g, corresponding to approximately 45% of the extracted arsenic (Grotti, Soggia, Goessler, Findenig, & Francesconi, 2010). As shown in Figure 5a,b, in the EH method, arsenic content of KO in the ethanol layer was almost sixfold that of the hexane layer, up to 12.84%, while fluorine content of KO in the hexane layer was greater than that in the ethanol layer (14.39% and 11.17%, respectively). Arsenic content in KO extracted with subcritical butane was also lower than that extracted via the subcritical butane‐dimethyl ether method (3.60%), while fluorine content displayed the opposite trend. Moreover, fluorine content of KO extracted via Folch method was the highest (14.91%).

**Figure 6:**
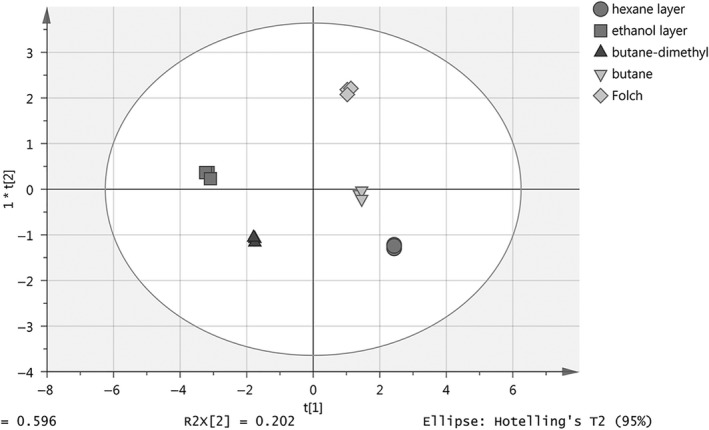
The classification effect of KO extracted through different methods. (Eight components)

As shown in Figure 6, through the OPLS‐DA analysis of the components in the KO, the KO of ethanol layer and that of hexane layer showed the largest difference, which proves the great separation effect of the EH method. In addition, the quality of KO extracted by subcritical butane‐dimethyl ether was similar to that of the ethanol layer, and the quality of KO extracted by subcritical butane was close to that of the hexane layer. The KO extracted through Folch method was more comprehensive.

In general, the quality of KO in the ethanol layer was competitive, but its high water content and FFA content would affect its shelf life. Further study should be conducted on how to reduce the water content of the KO in the ethanol layer.

## 4 CONCLUSIONS

In this study, KO was extracted via the EH method and subcritical method. Levels of PL, FA, and minor components including astaxanthin, FFA, fluorine, ash, and arsenic were compared. The results indicated that the EH method can be used to extract two kinds of KO with high nutritional value, simultaneously. The OPLS‐DA analysis demonstrated the great separation effect. The EH method yielded similar lipid content with the subcritical extraction method, and the antioxidant astaxanthin was abundant in KO extracted from the hexane layer. Meanwhile, KO in the ethanol layer had the highest levels of phospholipids *n*−3 PUFA and the lowest fluorine content, contributing to high nutritional value. This study not only provides novel approaches for valuable applications of Antarctic krill, but also a theoretical basis for the extraction and application of KO.

## CONFLICT OF INTEREST

The authors declare that they do not have any conflict of interest.

## ETHICAL STATEMENT

This study does not involve any human or animal testing. Written informed consent was obtained from all study participants.
