# Supplementary material for: The comparison of krill oil extracted through ethanol–hexane method and subcritical method
Source: Food Sci Nutr. 2019 Jan 28;7(2):700–10. doi: 10.1002/fsn3.914 (PMC6392833; doi:10.1002/fsn3.914)
Supplement: Supplementary file 1 [file FSN3-7-700-s001.docx]

**The optimization experiments:**

To determine the suitable volumetric ratio of ethanol:hexane, six volumetric ratios, 10:0, 8:2, 6:4, 4:6, 2:8, and 0:10 were set. 10 g of frozen Antarctic krill was weighed, and KO was extracted using EH method at room temperature (25℃) with a 1:25 solvent ratio. After 4 h of extraction, the filtrate was stratified, and the upper hexane layer and the lower ethanol layer were separated. KO in the upper and lower layers was obtained via rotary evaporation, then collected in a glass vessel for analysis.

To compare the effect of different reaction time, 10 g of frozen Antarctic krill was weighed, and the KO was extracted using 4:6 of ethanol/hexane at room temperature (25℃) with a 1:25 solvent ratio. After extracting for 1, 2, 3, 4, and 5 h, KO was extracted in glass vessels for analysis.

Moreover, to compare the effect of temperature, 20, 30, 40, 50, and 60°C were set up. 10 g of frozen Antarctic krill was weighed, and the KO was extracted using 4:6 of ethanol/hexane with a 1:25 solvent ratio. After 4 h of extraction, KO was extracted in glass vessels for analysis.

To determine the effect of the shrimp/solvent ratio on lipid extraction, five solvent volume gradients, 1:5, 1:15, 1:20, 1:25, and 1:30 were set up. 10 g of frozen Antarctic krill was weighed, and the KO was extracted using 4:6 of ethanol/hexane at room temperature (25℃). After 4 h of extraction, KO was extracted in glass vessels for analysis.

Scanning electron microscopy

The morphology of krill before and after EH extraction was assessed via scanning electron microscopy (SEM) (Hitachi S- 4800, Japan) at an accelerating voltage of 15 kV.

**The surface structure changes of Antarctic krill after extraction**

Surface morphology of krill powders before and after oil extraction was assessed via SEM. FigureS1a shows that before extraction, krill powder had a smooth surface and was covered with oily substances with no visible drops. After extraction, however, as shown in figureS1b, there were numerous voids on the surface of krill powder and some interstices were observed; furthermore, numerous muscle fibers were exposed. As the ratio of extraction efficiency was related to the amount and shape of the interstices in the samples, suggesting that solvents destroyed the outer structure of krill powder, thereby increasing the dissolution of oil.

| 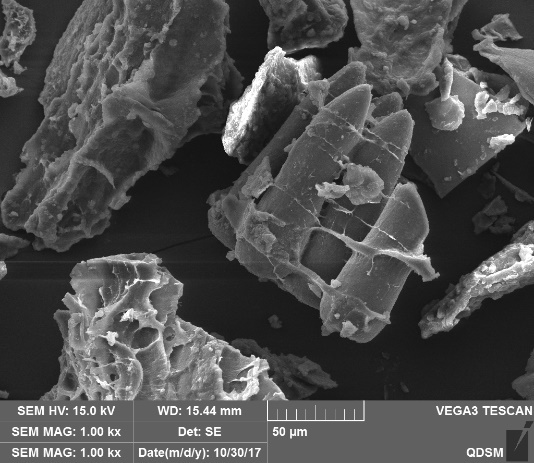a | 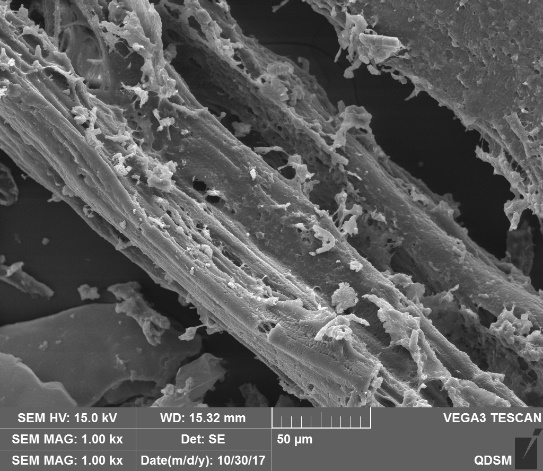b |
| --- | --- |

**FigureS1** Scanning electron micrographs of krill powders (a) before extraction and (b) after ethanol-hexane extraction.
